# Analysis of Patients Admitted for Asthma Exacerbation in a Tertiary Hospital in Spain

**DOI:** 10.7759/cureus.63042

**Published:** 2024-06-24

**Authors:** Eusebi Chiner, Clara Machetti, Ignacio Boira, Violeta Esteban, Carmen Castelló Faus, Anastasiya Torba Kordyukova

**Affiliations:** 1 Pulmonology, Hospital Universitario San Juan de Alicante, Alicante, ESP

**Keywords:** asthma exacerbation, treatment adherence, severe uncontrolled asthma, hospitalization, exacerbation, bronchial asthma

## Abstract

Objectives: To analyze the characteristics of adult patients admitted for asthma exacerbation and determine optimization, treatment adherence, and follow-up in clinics.

Methods: Patients ≥ 18 years old admitted from May 2021 to June 2023 with a primary diagnosis of asthma exacerbation were included. Patients with a secondary diagnosis of asthma exacerbation and those without a confirmed diagnosis were excluded.

Results: A total of 186 patients were analyzed, 63% were female, with a mean age of 49 ± 34 years, mean body mass index (BMI) of 26.4 ± 5 kg/m2, mean immunoglobulin E level of 132 ± 235 IU/mL (range: 25-2041), mean eosinophils count of 180 ± 443, and length of stay of 8.6 ± 5 days. Comparing patients with one admission to those with multiple admissions, differences were observed in age (39 ± 15 vs. 58 ± 20, p < 0.0001), BMI (25.2 ± 3 vs. 27.4 ± 4, p < 0.0003), comorbidity (15% vs. 60%, p < 0.0001), and length of stay (4.5 ± 2 vs. 11 ± 3, p < 0.0001). Of the patients, 15% had undiagnosed asthma, 28% had known asthma without maintenance therapy, 23% were managed by primary care, and 34% were followed by pneumology. The mean Test of Adherence to Inhalers (TAI) score was 42.5 ± 8 points, with 70% showing erratic non-adherence, 46% showing deliberate non-adherence, and 21% showing unconscious non-adherence.

Conclusions: The young population represents a significant percentage of admissions for asthma exacerbation due to poor follow-up in pulmonology clinics, inadequate treatment optimization, and low adherence. This study adds that it is necessary to improve the approach to asthma in primary care to optimize treatment, reduce under-diagnosis, and avoid hospital admissions.

## Introduction

Asthma has a prevalence of 5% in adults. It is estimated that between 5% and 10% of patients with asthma develop severe asthma. It is an inflammatory disease associated with bronchial hyperreactivity, with episodes of reversible bronchoconstriction triggered by allergens, exercise, or gastroesophageal reflux [1,2].

The AsmaCost study estimated that the global cost of asthma was 1.480 million euros per year. Of this expenditure, 70% was attributed to patients with poor control [3]. Therefore, international (Global Initiative for Asthma, GINA) and national (Spanish Guideline on the Management of Asthma, GEMA) guidelines propose alternative treatments to achieve and maintain disease control. The most promising treatments are biologic therapies, which target selective pathways [1,2].

Despite the increasing prevalence of asthma over the last three decades, there has been a gradual decrease in hospital admissions as the primary diagnosis and a reduction in asthma exacerbation diagnoses. Furthermore, there has been a reduction in the length of hospital stays and mortality rates [4]. However, when asthma is a secondary diagnosis during hospitalization, both increase significantly. This is likely due to an increase in comorbidities in the aging population [5].

It should be noted that exacerbations are common during the progression of the disease. Factors that increase the risk of prolonged hospitalization include female gender, advanced age, tobacco smoke, a history of severe exacerbations and previous admissions, the excessive use of short-acting beta-agonists (SABA) in the preceding 24 hours, inadequate treatment, or the winter season [1,4].

A primary objective in the management of asthma is the prevention of exacerbations. Approximately 27% of patients with well-controlled asthma and appropriate treatment develop therapeutic failure, and 15% will experience an exacerbation within a year. Moreover, readmissions occur predominantly in patients with more severe asthma, multiple comorbidities, poorer lung function, a greater number of emergency visits, and elevated blood eosinophil levels [6]. Furthermore, exposure to environmental pollutants has been linked to an increased risk of readmissions [7].

A number of modifiable factors in primary care contribute to poor asthma control. These include inappropriate prescriptions, poor treatment adherence, inadequate inhalation technique, persisting exposure to allergens, irritants, and comorbidities [8]. Inadequate management during the stable phase may occur due to the low rate of inhaled corticosteroids (ICS) employed and incorrect follow-up in primary care [7,9]. Only 61% of patients presenting to emergency departments with a previous diagnosis of asthma received ICS [10]. Additionally, the widespread use of SABA is associated with a higher risk of severe exacerbations (regardless of maintenance treatment) and oral corticosteroid use, which can lead to acute or chronic complications [11]. To prevent exacerbation in patients with poorly controlled asthma, it is essential to implement measures such as regulating the home environment, an appropriate follow-up in primary care, and referral of patients when necessary [12].

With regard to mortality, there was a period of elevated asthma mortality from the 1960s to the 1980s, which was attributed to the excessive use of SABA. This was followed by a decline in mortality rates as a result of the introduction of ICS, which achieved a 63% reduction in asthma mortality over the following 20 years. Nevertheless, there has been no further reduction in asthma mortality since that time [13]. Although the overall number of deaths has remained stable, there has been an increase in those over the age of 75. Potential explanations proposed include the chronic nature of the disease, diminished efficacy of medications, heightened susceptibility of the elderly to medication-induced side effects, and the possibility of some asthma-related fatalities being misclassified as due to other causes such as chronic obstructive pulmonary disease (COPD) [14].

The objective of this study was to evaluate the clinical and epidemiological characteristics of patients admitted for asthma, assess compliance with treatment guidelines, and estimate differences between patients with single hospital admission and those with more than one admission, as well as the type of care received from primary and specialized care.

## Materials and methods

Study type

This is a retrospective, observational, real-life study, conducted at a single center, based on the medical records of patients admitted for asthma exacerbation in the pulmonology department of a third-level university hospital serving a population of 250,000 people (Hospital Universitario San Juan de Alicante, Spain).

Study period

This study was conducted between January 2021 and January 2023 (24 months).

Population

The study included patients aged 18 years and above who had been diagnosed with asthma exacerbation. A systematic search was conducted in accordance with the International Classification of Diseases (ICD-10) for patients with codes J45.9, J46, J50, and other related codes as the primary diagnosis at discharge. An exacerbation was defined as a deterioration from the baseline requiring specific treatment [1].

The annual incidence per 100,000 people was calculated in relation to a reference population of 250,000 inhabitants. Moreover, the proportion of annual admissions to pulmonology for any respiratory cause and their seasonal distribution were determined. Asthma severity was defined in accordance with international guidelines based on the month preceding admission [1].

Patients whose primary diagnosis and reason for admission was not bronchial asthma exacerbation, even if this was included as a secondary diagnosis (asthma-COPD overlap syndrome, pulmonary embolism, community-acquired pneumonia, bronchogenic carcinoma, obstructive sleep apnea, etc.), were excluded.

Studied variables

Sociodemographic and anthropometric characteristics (age, sex, BMI), smoking status, asthma severity, atopic dermatitis, immunoglobulin E (IgE) levels, comorbidities (nasal polyposis, allergic rhinitis, gastroesophageal reflux disease (GERD), previous allergies, aspirin-exacerbated respiratory disease (AERD), obesity, obstructive sleep apnea (OSA), bronchiectasis, other eosinophilic diseases), and degree of control and compliance were included. These were evaluated using standardized definitions and confirmed through additional tests or validated questionnaires. The number of admissions per patient, type of follow-up (primary care/pulmonology), previous spirometry, prior treatment, and average hospital stay were assessed. Patients with a single hospital admission and those with two or more were analyzed to evaluate differences.

Instrumentation

The Asthma Control Test (ACT) [15] was employed. Higher scores (range = 20-25) are indicative of optimal control, while scores below 15 indicate inadequate control. Additionally, the Asthma Control Questionnaire (ACQ), comprising five questions scored from 0 to 6, was used [15].

The Test of Adherence to Inhalers (TAI) was employed to assess treatment adherence. This involved identifying patients with low adherence, determining the intensity of adherence, and guiding the type or pattern of non-compliance [16].

Spirometry was conducted according to the Spanish Society of Pulmonology and Thoracic Surgery (SEPAR) criteria, including reversibility tests [17].

Statistical analysis

The numerical variables representing the baseline and outcome were presented as the mean and standard deviation, respectively. The statistical significance of the observed differences was evaluated using either the Student's t-test (for unpaired data) or the Wilcoxon test, depending on the normality of the data. The chi-square test was used for qualitative variables. A p-value of less than 0.05 was considered to be statistically significant for all parameters recorded. All statistical analyses were performed using SPSS version 18 (SPSS Inc., Chicago, IL).

Ethical considerations

The study used anonymous data, and as it was a retrospective study, informed consent was not required. The protocol was approved by the Ethics and Research Committee with code 23/065.

## Results

A total of 2850 patients were admitted to the hospital during the study period, 186 of whom developed an asthma exacerbation (a total of 237 admissions). The incidence of asthma admissions per 100,000 population per year was 47/100,000.

**Figure 1:**
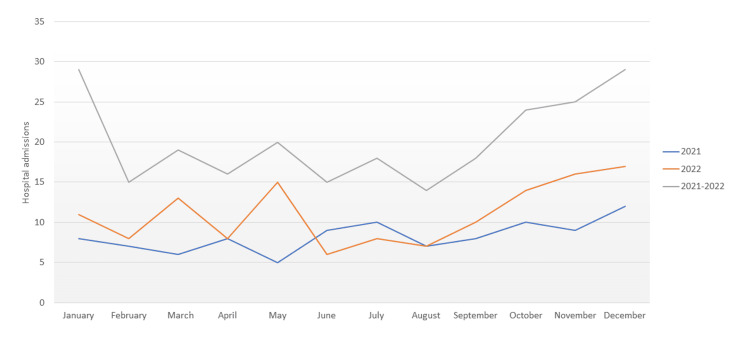
Seasonal variation in hospital admissions due to asthma exacerbation.

Figure 1 illustrates the monthly distribution of admissions disaggregated by year, with the resultant line for both years. It shows a slight increase in spring but a clear rise in the autumn-winter months.

Baseline patient characteristics are shown in Table 1. The most frequent comorbidities were rhinitis (66%), nasal polyposis (63%), anosmia/hyposmia (64%), atopic dermatitis (40%), AERD (18%), food allergy (6%), allergic bronchopulmonary aspergillosis (6.5%), OSA (4%), obesity (6%), bronchiectasis (2%), and other eosinophilic diseases (1.6%).

**Table 1:** Baseline characteristics of the patients.

Variable	
Sex, n (%)	108 females (58%)/78 males (42%)
Age, mean ± SD	49 ± 34 years
BMI, mean ± SD	26.4 ± 5 kg/m^2^
Immunoglobulin E, mean ± SD	132 ± 235 IU/mL
Eosinophils, mean ± SD	180 ± 443 cells/mcL
Comorbidity, n (%)	143 patients (77%)
Current smokers, n (%)	33 patients (18%)
Emotional disturbance, n (%)	52 patients (28%)

The most common triggers of exacerbation were acute respiratory infection in 120 patients (65%) being the most common etiology, *Influenza B virus* infection in 18 patients, respiratory syncytial virus (RSV) in 15, *Streptococcus pneumoniae* in 12, *Haemophilus Influenzae* in seven, *Chlamydia pneumoniae* in six, with the rest of unknown infectious etiology; allergen exposure was present in 26 patients (14%) (18 perennial and eight seasonal allergies), unknown cause in 22 patients (12%), poor response to treatment in 15 patients (8%), and acetylsalicylic acid and other nonsteroidal anti-inflammatory drugs (NSAID) ingestion in two patients (1%).

Previously diagnosed asthma patients were prescribed one or more of the following as routine medication: low/high doses of inhaled corticosteroids/long-acting beta 2-agonists (ICS/LABA) in 186 patients (100%), montelukast in 84 patients (45%), long-acting anticholinergics (LAMA) in 89 patients (48%), antihistamines in 28 patients (15%), xanthines in 56 patients (3%), and oral steroids in 15 patients (8%). Twenty patients (3%) were undergoing biological treatment (a total of 10 patients were treated with omalizumab, seven with mepolizumab, and three with benralizumab), and 54 patients (29%) had received at least one course of steroids in the past year. A total of 15 patients received allergen immunotherapy, with only 35% (42 patients) receiving the influenza vaccine and 21% (25 patients) the pneumococcal vaccine.

Twenty-eight patients (15%) had their first hospital admission for previously unknown asthma. The severity level of known asthma patients (n = 158) is presented in Table 2. With regard to control status, patients exhibited an ACT score of 17 ± 2 points and an ACQ score of 1.5 ± 1, indicating overall poor asthma control.

**Table 2:** Severity level and GEMA step of patients with known asthma. GEMA: Spanish Guideline on the Management of Asthma.

Asthma severity	GEMA step	N	%
Mild	1	5	3
Mild persistent	2	20	13
Moderate persistent	3-4	101	64
Severe persistent	5-6	32	20

Table 3 illustrates that 150 patients (81%) were admitted only once, while 36 patients (19%) were admitted two or more times. Some patients were admitted up to five times. Additionally, five patients required admission to the intensive care unit (ICU) due to severe crisis, with one requiring invasive mechanical ventilation. However, there were no fatalities.

**Table 3:** Distribution of patients by number of admissions.

Number of patients	Hospital admissions	%
150	150	81
36	87	19
17	2	9
14	3	8
4	4	2
1	5	1

Table 4 presents a comparison of the characteristics between patients with a single admission and those with multiple admissions. Rhinitis, nasal polyposis, and anosmia/hyposmia were the most common comorbidities in patients with multiple admissions.

**Table 4:** Comparison between patients with only one hospital admission versus those with more than one admission.

	1 admission	>1 admission	t-value/X^2^	P
Age (mean ± SD)	39 ± 15	58 ± 20	T-value = 8.293	<0.0001
Gender	89 women/61 men	28 women/8 men	X^2 ^= 3.479	0.06
BMI (mean ± SD)	25.2 ± 3	27.4 ± 4	T-value = 4.801	<0.0003
Comorbidity	15%	60%	X^2 ^= 49.595	<0.0001
Average hospital stay (mean ± SD)	4.5 ± 2	11 ± 3	t-value = 19.976	<0.0001

The mean length of stay was 8.6 ± 5 days. A total of 72 patients (39%) were over the age of 65 years at the time of admission. Table 5 displays the number of patients with unknown asthma, known asthma without maintenance treatment, patients only managed by primary care, or those managed in pneumology. Significant differences were observed in the percentage of patients with prior lung function testing between those from primary care and pneumology (chi-square = 60.095, p < 0.0001). A total of 96 patients (52%) had undergone spirometry prior to the study, with a mean forced expiratory volume percentage in one second (FEV1%) of 78 ± 22%.

**Table 5:** Characteristics of the degree of diagnosis and type of follow-up of the patients, as well as the performance of previous spirometry.

	Patients with unknown asthma	Patients with known asthma without maintenance treatment	Patients with known asthma controlled by primary care only	Patients already controlled in pulmonology office
Number (%)	28 (15%)	52 (28%)	42 (23%)	64 (34%)
Spirometry	0	22 (42%)	10 (24%)	64 (100%)

Table 6 presents the level of compliance as measured by the TAI questionnaire in patients with a previous diagnosis of asthma. The mean score on the TAI (out of 10 items) was 42.5 ± 8 points. A total of 28% of the sample (n = 44) exhibited good adherence, scoring 50 points. The level of adherence was classified as intermediate (46-49 points) in 26% (n = 41) and poor (≤45 points) in the remainder (46%, n = 73). The overall prevalence of non-adherence, as determined by the TAI (≤49 points), was 72%. By gender, the prevalence of non-adherence was 75% in females and 72% in males (p = ns).

**Table 6:** The Test of Adherence to Inhalers (TAI) scores.

	n = 158	%
TAI, 10 items (mean ± SD)	42.5 ± 8	
Good adherence (50 points)	44	28
Low adherence (≤49 points)	114	72
Intermediate adherence (46-49 points)	41	26
Poor adherence (≤45 points)	73	46
TAI, 12 items (mean ± SD)	47 ± 6	
Erratic non-compliance		
No	47	30
Yes	111	70
Deliberate non-compliance		
No	85	54
Yes	73	46
Unconscious non-compliance		
No	125	79
Yes	33	21

Regarding the type of non-compliance identified by the TAI test (12 items), 111 patients (70%) exhibited erratic non-compliance, defined as items 1-5 scoring below 25 points, 73 patients (46%) demonstrated deliberate non-compliance, defined as items 5-10 scoring below 25 points, and 33 patients (21%) evidenced unconscious non-compliance, defined as items 11-12 scoring below four points.

## Discussion

The study findings indicate that asthma exacerbations represent a substantial proportion of admissions to pulmonology (8.3%), with an admission incidence of 47/100,000 inhabitants per year when considered as the primary diagnosis. This incidence is lower than that reported in the Basque Country (88.9%) [18] and is similar to the trends observed in Spain from 2011 to 2020 [5].

In our study, the highest number of admissions for asthma as the primary diagnosis was observed in young patients, although 40% were aged over 65 years, as several studies have indicated [5,19]. Most patients with multiple admissions belong to the older age group. Factors contributing to increased admissions among the elderly include comorbidities, treatments that may exacerbate asthma, and greater lung function deterioration due to undertreatment. A recent study indicated that exacerbations are associated with multimorbidity, asthma severity, poor control, increased obstruction, and a high inflammatory pattern [20]. The higher seasonality observed in the autumn and winter months is related to respiratory infections, as evidenced in other series [5]. However, pollen exposure is also a risk factor for moderate to severe exacerbations, particularly in individuals under the age of 18 years [21].

Readmissions are influenced by the patient or underlying disease characteristics. A high readmission rate is observed in the month following discharge for asthma. Over periods of follow-up ranging from one to 11 years, it has been observed that between 16% and 25% of patients experienced new hospital admissions [4].

It is important that 15% of patients were unaware they had asthma, and 28% of those with known asthma were not on maintenance treatment, only using SABA. Furthermore, a considerable proportion of patients lacked prior lung function measurements (48%), and there was a low proportion of spirometry tests in patients followed up by primary care. The overuse of SABA and underuse of ICS have been associated with poor asthma control, an increased risk of hospitalization, and asthma mortality [22].

In our series, instances of poor adherence to GEMA guidelines and low compliance with TAI are significant factors in the exacerbation of asthma. Both patients with severe and non-severe asthma tend to underestimate the severity of their condition, which is a key factor in the development of exacerbations [23].

In our study, the number of patients with a history of biological treatment is relatively low, reflecting the fact that this treatment has the effect of reducing admissions and resource consumption [1]. Nevertheless, patients with severe asthma may still be admitted to the hospital despite receiving biological treatment. However, there is a tendency for this to improve over time compared to previous years. The advent of biological therapies has brought about significant changes in the management of exacerbating patients, resulting in a reduction in admissions and readmissions [24]. In our study, the majority of admissions were attributable to neutrophilic exacerbations, which were either caused by infections or poor compliance, as indicated by the TAI questionnaire.

From 2011 to 2020, over 1.1 million adults in Spain were hospitalized with asthma. The proportion of men and women aged ≥65 increased over time, while the proportion of younger individuals decreased. As observed in our study, women constituted 72% and men constituted 28% of the study population [25]. A higher percentage of women has been reported consistently, ranging from 61.14%, as reported by De Miguel-Díez et al., to 74.5%, as reported by González-Barcala et al. [25,26]. The factors that contribute to this phenomenon include the higher prevalence of asthma in adult women and the difference in symptom intensity for the same severity level. A study of patients who had been hospitalized for asthma and were on treatment found that females exhibited more symptoms and a worse quality of life but better lung function [27]. Furthermore, they are at a higher risk of exposure to household and workplace irritants [4]. Other contributing factors include their longer life expectancy and higher prevalence of anxiety and depression, which are associated with increased use of healthcare services [4].

In Western countries, there is a trend toward a reduction in the number of hospital admissions for asthma. In 2020, a decrease in asthma exacerbations was observed in numerous countries, coinciding with the onset of the COVID-19 pandemic. The precise causes of this reduction in hospital admissions for asthma remain unclear. However, it is possible that handwashing, mask-wearing, and social distancing may have reduced exposure to triggers such as infections and allergens. Additionally, it is postulated that inhaled corticosteroids confer protection against SARS-CoV-2 infection and the development of severe diseases [28]. A recent study conducted in the UK revealed an increase in asthma exacerbations following the relaxation of measures implemented to combat the spread of the novel COVID-19, which was associated with an increase in respiratory infections [29].

A similar decline in admissions was also observed in our hospital. Conversely, the prevalence of admissions per 100,000 was comparable to that observed prior to the pandemic.

Another essential aspect of hospital admissions is their substantial influence on disease-related costs. The AsmaCost study estimates the overall cost of asthma to be 1.480 million euros per year. A total of 70% of this expenditure is attributed to patients with poor control [3].

The findings of our study indicate that readmitted patients are older, with higher rates of obesity and comorbidities, and consume more resources, resulting in longer hospital stays. The average length of stay in Spain is approximately seven days, with a tendency to decrease. This is attributed to improved management of exacerbations. Nevertheless, the group with more comorbidity presents prolonged stays [29].

It is notable that, in our study, there were no asthma-related deaths. However, 2.7% of admissions were to the ICU, with one patient (0.5%) requiring invasive mechanical ventilation. The trend in asthma mortality in Spain has shown a progressive decrease from 7.38 to 2.03 deaths per 100,000 from 1980 to 2019 [30].

The limitations of our study derive from the fact that it is a period of only two years, which precludes the establishment of trends such as mortality or the evaluation of the decrease in admissions over a long period of time. However, we carried out a systematic collection of real-life data with results that are highly consistent with those of the main existing studies. This allows us to make decisions in the approach to asthmatic patients, with the aim of improving the relationship between primary medicine and specialized asthma units.

It is crucial to identify patients at risk of asthma exacerbation in primary care, particularly young individuals without maintenance treatment or with low compliance. Additionally, monitoring older patients (>65 years) and comorbidities, as well as increasing the performance of pulmonary function tests to prevent admissions for asthma exacerbation is important.

## Conclusions

It can be concluded that a considerable number of cases of asthma could be prevented if optimal treatment was provided in accordance with clinical practice guidelines, and adherence to treatment protocols was enhanced. The use of spirometry in primary care is essential to detect asthma in its early stages and thus prevent its worsening. Patients with comorbidities, particularly in advanced age, require close monitoring. Finally, our study examines the epidemiology of asthma exacerbations in our setting and identifies risk factors to facilitate appropriate collaboration between primary care and hospital care.
